# Collective case study on periodized training in elite female middle- and long-distance swimmers preparing for national competition

**DOI:** 10.3389/fphys.2026.1943799

**Published:** 2026-10-27

**Authors:** Tianyi Gao, Jiawen Li, Xinjia Shan, Jiayi Lyu, Dongxiang Huang, Jie Gao

**Affiliations:** 1 School of Education, Beijing Sport University, Beijing, China; 2 Yale College, Yale University, New Haven, CT, United States; 3 School of Physical Education, Shaoguan University, Shaoguan, Guangdong, China

**Keywords:** altitude training, collective case study, elite swimmers, periodized training, training intensity distribution

## Abstract

**Background:**

Elite swimmers increasingly face calendars requiring multiple performance peaks within a single season, a scenario for which traditional single-target periodization models may be insufficient.

**Methods:**

This longitudinal collective case study followed five elite female middle- and long-distance freestyle swimmers across a 52-week macrocycle preparing for two national championships. The program combined three altitude camps, sea-level integration phases and two timed tapers. Training intensity distribution (TID) was quantified with a three-zone lactate-threshold model; hemoglobin, hematocrit, red blood cell count, creatine kinase and cortisol were measured at four time points; and performance was assessed by time-trial and official competition results. Given N = 5, analysis prioritized a smallest-worthwhile-change (SWC) classification for performance and paired effect sizes with 95% confidence intervals for blood markers rather than null-hypothesis significance testing. Hematological outcomes are concentration-based indices; training-load descriptors refer to in-water swimming only.

**Results:**

Applying an SWC of ≈0.24% to the 20 athlete-by-event observations, 12/20 (60%) changes at Competition 1 and 18/20 (90%) at Competition 2 exceeded the SWC, most representing improvement over baseline; this ordering held across the full defensible range of reference variability (SWC 0.22–0.42%). Hematological effect sizes were largest at Week 27 (RBC: Hedges’ g = 2.32, 95% CI [0.64, 4.01]; HCT: g = 2.04 [0.50, 3.58]), while creatine kinase and cortisol peaked at Week 17 and were closer to baseline at Week 49, compatible with a lower physiological stress state at that sampled time point. TID shifted across phases (pyramidal during base development, polarized during altitude blocks, threshold-oriented during race-pace sharpening), with marked inter-individual heterogeneity in performance and physiological responses.

**Conclusion:**

Across this double-peak macrocycle, most athlete-by-event changes exceeded the SWC, and the proportion doing so was higher at the second championship than at the first; these changes occurred alongside altitude-associated increases in concentration-based oxygen-transport indices and a taper-associated normalization of creatine kinase and cortisol. With no control group or randomization, only five athletes and four blood samples, several simultaneous interventions and unmeasured confounders (iron status, menstrual-cycle phase, dryland load), these findings describe associations only. They are offered as exploratory, hypothesis-generating observations from a single elite squad rather than as causal evidence.

## 1 Introduction

Periodized training is a foundational principle in elite sport: training load, content, and recovery are systematically sequenced so that athletes arrive at peak physiological and psychological readiness for targeted competitions (Kiely, 2018). Annual plans are typically organized into macrocycles, mesocycles, and micro cycles, each with its own training objectives and load characteristics (Hermosilla et al., 2021). In swimming, traditional periodization begins with high-volume, low-intensity work and progressively shifts toward lower-volume, higher-intensity training, whereas reverse periodization inverts this sequence (Clemente-Suárez et al., 2017; Clemente-Suárez et al., 2018); comparative studies indicate that both approaches can produce meaningful physiological adaptation (Clemente-Suárez et al., 2017). Block-periodization models are further differentiated into Concentrated-Unidirectional and Multi-Targeted approaches; evidence suggests the multi-targeted model holds greater promise for elite swimmers, although its application to race-specific peaking requires further investigation (Issurin, 2016). A competitive season is commonly structured around preparatory, specific preparation, pre-competition, and competition phases, with training volume and intensity adjusted according to the athlete’s competition calendar and adaptive status (Hermosilla et al., 2021).

Training intensity distribution (TID) describes how training time or volume is allocated across physiological intensity zones, typically delineated by heart rate, power, velocity, or percentage of VO_2max_ (Seiler, 2010; Campos et al., 2021; Sun et al., 2025). Swimming research commonly adopts a three-zone model anchored to the first and second lactate thresholds (Seiler and Kjerland, 2006): Zone 1 (below the first threshold, blood lactate < 2 mmol·L^-^¹), Zone 2 (between the two thresholds, ≈4 mmol·L^-^¹), and Zone 3 (above the second threshold, > 4 mmol·L^-^¹). Three canonical TID patterns are described in the endurance literature: a pyramidal model, in which most volume sits in Zone 1, favored by many elite endurance athletes; a polarized model, combining a very high Zone 1 proportion with substantial Zone 3 work while minimizing Zone 2, generally regarded as an effective stimulus for enhancing performance in already well-trained athletes (Stöggl and Sperlich, 2014); and a threshold model, concentrating volume in Zone 2, whose effectiveness appears more modest than the polarized approach in direct comparison (Pla et al., 2019).

Altitude training is now integral to periodized preparation in elite swimming, with a growing evidence base linking it to both performance gains and hematological adaptation (Astridge et al., 2024; Carin et al., 2025), although individual hematological responses to a given altitude stimulus vary considerably (Carin et al., 2025). Blood-based indicators — hemoglobin, hematocrit, red blood cell count, creatine kinase, and cortisol — are widely used in swimming to track physiological status, training load, and recovery, supporting evidence-based adjustment of training plans and injury prevention (Pollock et al., 2019). Hemoglobin and hematocrit reflect oxygen-carrying capacity, with changes potentially signaling training adaptation, iron deficiency, or overtraining (Li et al., 2015); elevated creatine kinase indicates muscle microtrauma, and persistently high values may reflect inadequate recovery (Brancaccio et al., 2007; Rahmanian et al., 2022); cortisol, a stress hormone, can signal overtraining and immune suppression when chronically elevated (Qiu et al., 2018).

While existing research has systematically examined periodization models, TID patterns, and hematological monitoring in swimming, this evidence base has focused predominantly on preparation for single-target competitions (González-Ravé et al., 2021). For elite athletes who must successfully navigate two or more major events within a single season, the periodized training strategies, physiological adaptation trajectories, and peak-performance regulation involved remain comparatively underexplored — including how TID is dynamically adjusted across a multi-peak season, how key hematological indicators fluctuate throughout, and how consecutive competitive performances actually unfold. A further, largely separate gap concerns statistical practice: because elite-athlete cohorts are inherently small, much of this literature must choose between under-powered group-level significance testing and purely narrative case description, and explicit guidance on a middle path — quantifying individual change against known measurement variability while still reporting effect sizes and confidence intervals — is not yet routine in this specific applied context (Halperin, 2018; Ruddock et al., 2019).

The specific contribution of the present study can therefore be stated directly. Training intensity distribution, altitude exposure, tapering and hematological monitoring in elite swimming have each been characterized in recent work (Pla et al., 2019; González-Ravé et al., 2021; Hermosilla et al., 2021; Astridge et al., 2024; Mujika et al., 2024; Carin et al., 2025), but each of those studies describes one component, over one training block, within one preparation. The present study reports a full competitive year in a single elite squad, and its contribution rests on three features of that record. First, it resolves training intensity distribution week by week across the whole macrocycle, documenting deliberate within-season switching between pyramidal, polarized and threshold models matched to the function of each block — a sequential, purpose-matched use of all three that recasts the comparison between them as a question of placement within a season rather than of categorical superiority. Second, it follows three altitude camps within one season, with blood sampling positioned around each of them, so that the hematological trajectory described is the cumulative one produced by repeated hypoxic exposure across a year rather than the response to a single camp. Third, it does so across a genuine double-peak season — two national championships 23 weeks apart within one uninterrupted macrocycle — in elite female middle- and long-distance swimmers, with every outcome reported at the level of the individual athlete and event and derived from official competition rather than laboratory time-trials.

To address these gaps, the present study conducted a longitudinal collective case study, systematically tracking five elite female middle- and long-distance swimmers preparing for two major national competitions within a single season. The objectives were threefold: to describe the training arrangement and periodization structure used across the season; to characterize the dynamic adjustment of training intensity distribution within a periodized plan designed for two successive competitions; and to quantify changes in key hematological indicators and competitive performance across training phases, using an analytic framework whose statistical claims are calibrated to what a five-athlete case series can actually support.

## 2 Methods

### 2.1 Study design

A longitudinal collective case study design was used, in which five elite athletes were prospectively followed across a single 52-week macrocycle culminating in two national championships. Case-based designs of this kind are increasingly employed in elite-sport research because randomized controlled trials are frequently impractical in populations of world-class athletes, where squad sizes are inherently small and training content cannot ethically or logistically be manipulated for experimental purposes (Ruddock et al., 2019). Formal causal inferences about the effectiveness of a specific training intervention cannot be drawn from a case series, and such designs remain susceptible to selection and observation bias (Ruddock et al., 2019); nevertheless, their principal strength lies in the depth of individual-level analysis they permit, which helps bridge the recognized gap between research evidence and applied coaching practice (Halperin, 2018). A structured, multidisciplinary approach to data collection — combining standardized performance testing with serial biological monitoring — was therefore adopted to maximize the scientific rigor attainable within a single-cohort, non-controlled framework (Sands et al., 2019). This design mirrors previous collective case-study work characterizing periodized altitude training in high-level swimmers (González-Ravé et al., 2023), providing methodological precedent for the present investigation.

### 2.2 Participants

Five elite female freestyle swimmers specializing in the 200 m to 1500 m events were enrolled, comprising four athletes of international-level Master of Sports status and one athlete of national-level Master of Sports status, all in preparation for major domestic competitions. Descriptive characteristics (mean ± SD) were age 20.00 ± 3.67 years, height 169.4 ± 2.88 cm, and body mass 56.75 ± 2.63 kg. Competitive standard, quantified using World Aquatics Points (formerly FINA Points), averaged 858.60 ± 43.47 (200 m), 866.60 ± 48.18 (400 m), 853.20 ± 62.89 (800 m), and 838.80 ± 59.47 (1500 m freestyle). The study protocol conformed to the principles of the Declaration of Helsinki and was approved by the Ethics Committee of the School of Physical Education and Sports Science, South China Normal University (Approval No. SCNU-SPT-2026-009). Written informed consent was obtained from all athletes, from the legal guardians of athletes under 18 years of age, and from their coach prior to data collection.

### 2.3 Periodized training intervention

Phase 1 (Weeks 1–19). The macrocycle began with an 8-week sea-level block (Sea-level I) to re-establish general aerobic capacity and training tolerance after the off-season, with a fasting venous baseline blood sample obtained at 6:30 a.m. on the Monday of Week 7. A 7-week altitude camp followed (Altitude I, 1890 m) to stimulate erythropoiesis and aerobic metabolic efficiency through chronic hypoxic exposure, before a 4-week sea-level integration block (Sea-level II) converted these adaptations into normoxic swimming performance; a second blood sample was collected at Week 17, and a benchmark time-trial was performed in Week 18.

Phase 2 (Weeks 20–28). Preparation for Competition 1 comprised a 6-week altitude camp (Altitude II, 2400 m) targeting event-specific race-pace work, followed by a 3-week sea-level taper (Sea-level III) to reduce accumulated fatigue and sharpen power output. A third blood sample was drawn at Week 27, ahead of competition in Week 29.

Phase 3 (Weeks 30–51). The final phase opened with an 8-week sea-level block (Sea-level IV, Weeks 30–37) for technical re-stabilization, followed by a 10-week altitude block (Altitude III, 1890 m, Weeks 38–47) constituting the principal training stimulus for the season’s final peak, and concluded with a 4-week sea-level taper (Sea-level V, Weeks 48–51) calibrated for supercompensation. A fourth blood sample was collected at Week 49, two weeks after the end of the final altitude block, ahead of the season’s main championship in Week 52 (**Figure 1**).

**Figure 1:**
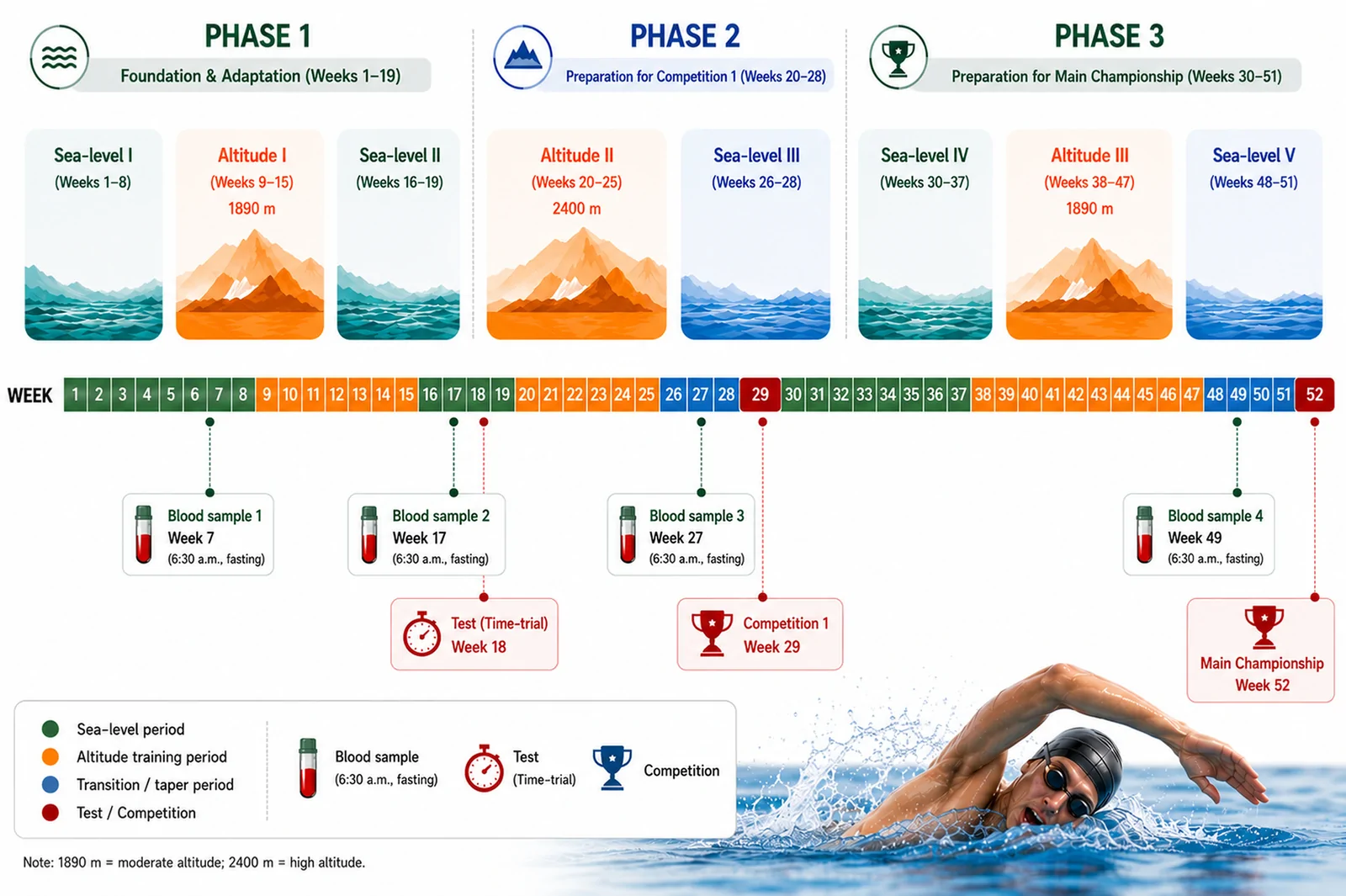
Timeline of the periodized training phases.

Altitude exposures of 1800–2500 m were selected because this range is consistently reported to elicit measurable increases in total hemoglobin mass and red-cell indices in elite swimmers while limiting the ventilatory, sleep, and recovery costs associated with higher elevations (Mujika et al., 2024), although marked inter-individual variability in the hematological response to altitude exposure has been documented (Carin et al., 2025). The overall taper-and-peak structure across the three phases followed established load-modeling principles for the weeks preceding major competitions in elite swimmers (Hellard et al., 2017).

**Figure 1** is a schematic illustration of the study design and was produced with the assistance of a generative artificial intelligence image-generation tool: ChatGPT Images 2.0 (image-generation model gpt-image-2; OpenAI, San Francisco, CA, USA; https://chat.openai.com), accessed 7 August 2026. The tool was used exclusively for visual rendering and layout of the diagram. All information depicted in the figure — the training phases and their week ranges, the altitude elevations, the blood-sampling weeks, and the time-trial and competition time points — was specified by the authors and was verified against the study protocol after generation. **Figure 1** contains no measured data. **Figures 2**, **3** and **4** were generated directly from the study dataset in Python using Matplotlib (version 3.10.9; https://matplotlib.org) and involved no generative AI. Full details are given in the Acknowledgements.

**Figure 2:**
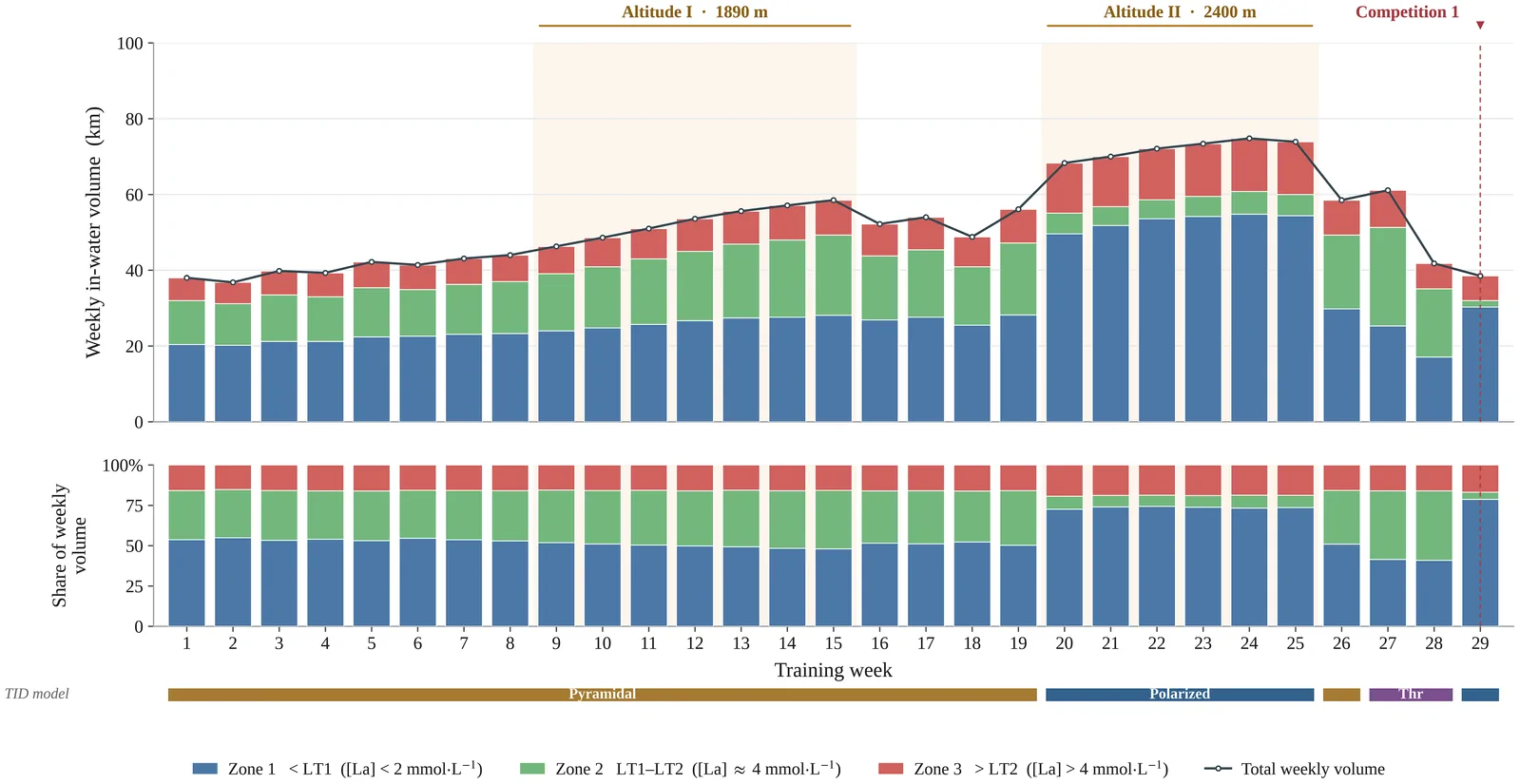
Weekly in-water training volume and training intensity distribution, Weeks 1–29 (Phase 1 and Phase 2).

**Figure 3:**
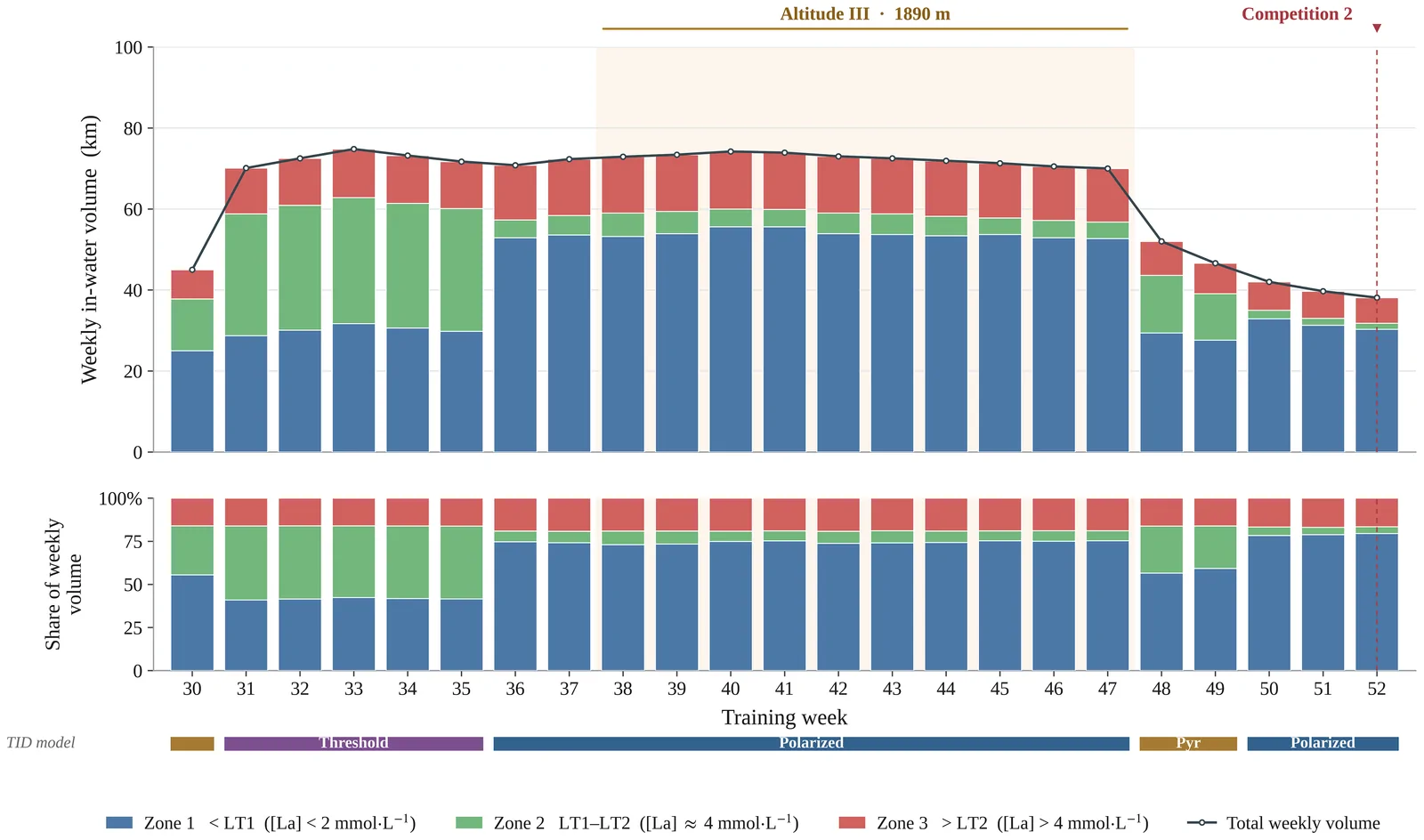
Weekly in-water training volume and training intensity distribution, Weeks 30–52 (Phase 3).

**Figure 4:**
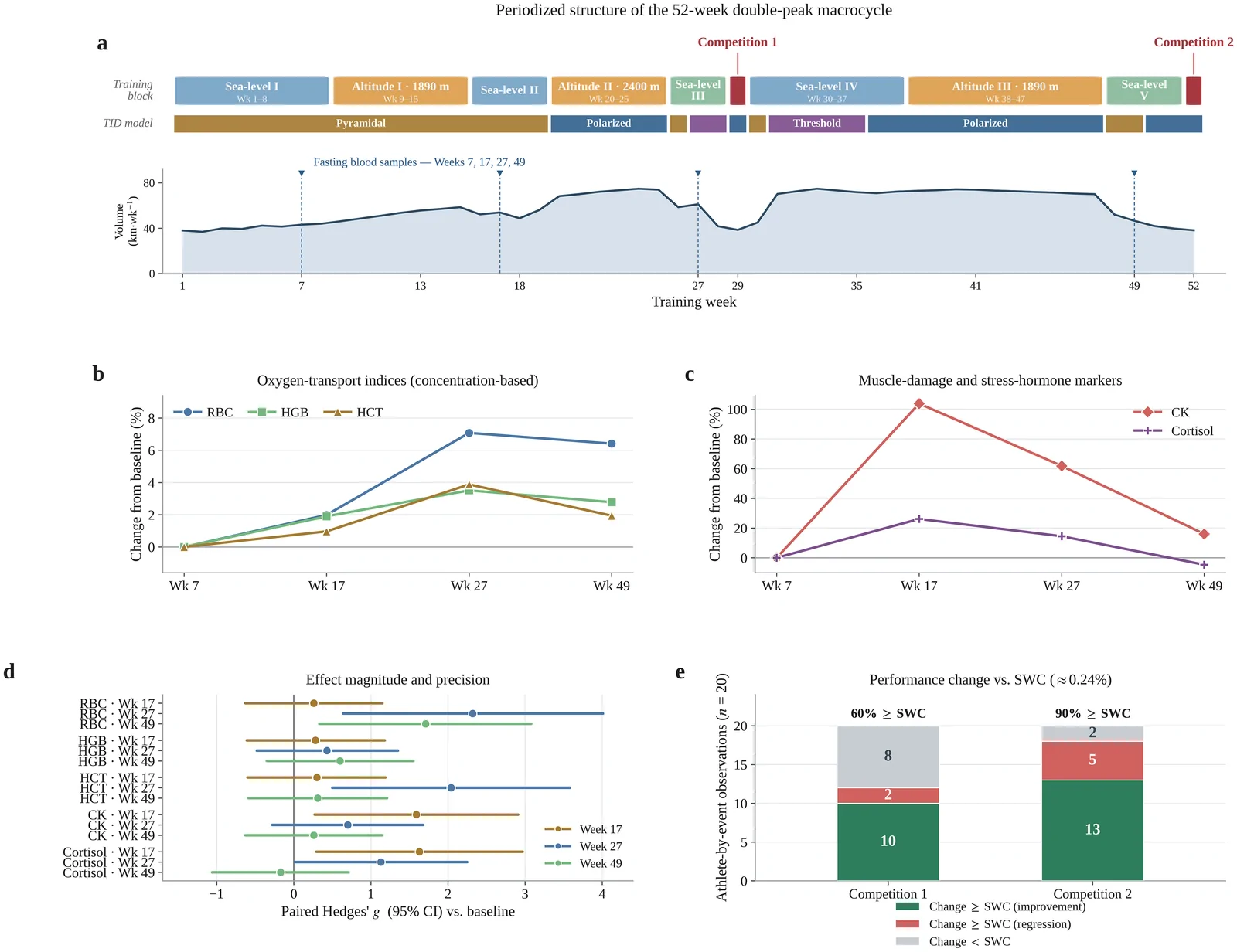
Graphical summary of the interaction between periodization, altitude exposure, hematological response and competitive performance across the 52-week double-peak macrocycle. **(a)** Periodized structure of the macrocycle: training blocks, dominant training-intensity-distribution (TID) model, weekly in-water training volume, fasting blood-sampling weeks (Weeks 7, 17, 27 and 49) and the two championships. **(b)** Group-mean percentage change from baseline in the concentration-based oxygen-transport indices (RBC, HGB, HCT). **(d)** Group-mean percentage change from baseline in creatine kinase (CK) and cortisol. **(d)** Paired Hedges' g (95% CI) versus baseline for each marker at Weeks 17, 27 and 49. **(e)** Classification of the 20 athlete-by-event performance changes against the smallest worthwhile change (SWC, approx. 0.24%) at Competition 1 and Competition 2.

### 2.4 Outcome measures

Swimming performance. Race performance was assessed at three time points: a middle- and long-distance freestyle time-trial (Week 18), Competition 1 (Week 29), and Competition 2 (Week 52), using each athlete’s time in her primary events (200 m, 400 m, 800 m, 1500 m freestyle).

Hematological and biochemical monitoring. Venous blood (4 mL) was collected from the antecubital vein under standardized fasting conditions at 6:30 a.m. on four occasions — Weeks 7, 17, 27, and 49 — consistent with recommended practice for longitudinal biological monitoring of elite athletes (Sands et al., 2019). Hemoglobin and hematocrit were assayed as indices of oxygen-carrying capacity and training adaptation (Li et al., 2015); creatine kinase, a validated marker of exercise-induced muscle damage and recovery status (Brancaccio et al., 2007; Rahmanian et al., 2022), and cortisol, reflecting cumulative training-stress and neuroendocrine load (Qiu et al., 2018), were assayed alongside red blood cell count to characterize the physiological cost of each training phase.

Standardization and sample handling. All four samples were drawn at 06:30 h on the Monday of the sampling week, after an overnight fast of ≥ 10 h and before any training that day. Each sampling Monday followed the squad’s scheduled recovery day, so that ≥ 24 h had elapsed since the last session and no maximal, exhaustive or unaccustomed resistance session had been performed within the preceding 48 h; athletes refrained from alcohol and caffeine for 24 h and rested seated for 10 min before venipuncture, standardizing the postural component of plasma-volume variation across the four time points. Each 4 mL draw was split into a 2 mL K_2_EDTA tube (complete blood count, analyzed within 2 h) and a 2 mL serum separator tube (clotted 30 min, centrifuged at 1500 × g for 10 min at 4 °C, analyzed the same day). Red blood cell count, hemoglobin and hematocrit were measured on an automated hematology analyzer (Sysmex XN-1000, Sysmex Corporation, Kobe, Japan), hematocrit being derived by cumulative red-cell pulse-height detection rather than by centrifugation; creatine kinase was determined by an IFCC-traceable enzymatic kinetic method (37 °C, N-acetylcysteine-activated) on a cobas c 702 analyzer and cortisol by electrochemiluminescence immunoassay on a cobas e 801 module (both Roche Diagnostics, Mannheim, Germany). All four occasions were assayed in the same laboratory by the same methods, internal quality control was within acceptance limits throughout, and laboratory inter-assay coefficients of variation were < 2% for the hematological indices, < 3% for creatine kinase and < 5% for cortisol. The samples therefore reflect a rested, standardized state rather than an acute post-exercise response — a distinction that matters most for creatine kinase and cortisol.

Sampling frequency. The four samples were separated by 10–22 weeks, an interval dictated by the camp and competition schedule. This resolution characterizes the phase-level physiological state on entering and leaving each major training block; it cannot resolve within-block kinetics, and the consequences differ by marker, since red-cell indices turn over across weeks to months whereas creatine kinase peaks 24–72 h after a damaging session and cortisol varies markedly within and between days.

Determination of individual lactate thresholds. LT1 and LT2 were determined from a discontinuous incremental step test in a 50 m pool: 7 × 200 m front crawl beginning at approximately 70% of each athlete’s current 400 m race pace and increasing by approximately 2.5% of that pace at each step, with the final step performed maximally and 1 min of passive recovery between steps. A 20 µL end-to-end capillary sample was taken from the fingertip after each step, transferred into a hemolyzing reagent tube and analyzed poolside (Biosen C-Line, EKF Diagnostic GmbH, Barleben, Germany), the analyzer being calibrated against a 12 mmol·L^-^¹ standard before each session; pace was controlled by the coach with split feedback every 50 m. Blood lactate was plotted against mean 200 m velocity and the thresholds derived by fixed-concentration criteria with linear interpolation between adjacent steps: LT1 at the first sustained rise above resting values (≈ 2 mmol·L^-^¹) and LT2 at 4 mmol·L^-^¹. Testing was performed in Week 1, repeated in Week 19 before departure for the Phase 2 altitude camp and again in Week 30, and the velocity boundaries used to classify training load were updated after each test. All three tests were conducted at sea level and the sea-level boundaries were applied unchanged during the altitude blocks, so zone allocation at altitude is correspondingly approximate (§4.6).

Training-load quantification and intensity distribution. Training load was classified from swimming velocity and its corresponding blood lactate concentration, using a three-zone model referenced to individually determined lactate thresholds (Seiler and Kjerland, 2006; Stöggl and Sperlich, 2014): Zone 1, velocities below the first lactate threshold (blood lactate < 2 mmol·L^-^¹); Zone 2, velocities between the first and second thresholds (blood lactate ≈ 4 mmol·L^-^¹); and Zone 3, velocities above the second threshold (blood lactate > 4 mmol·L^-^¹). Split velocities from every training session were assigned to the corresponding zone, and TID for each macrocycle phase was expressed as the percentage of total training volume completed in each zone. The dominant TID pattern per phase was then classified as pyramidal (Z1 > Z2 > Z3), polarized (very high Z1, comparatively high Z3, minimal Z2), or threshold-oriented (Z2-dominant).

### 2.5 Statistical analysis

With five athletes, formal null-hypothesis significance testing is not an informative option: a nonparametric repeated-measures test cannot reach conventional thresholds however large the true effect, and a mixed-effects model with five athlete-level clusters gives an unstable estimate of between-athlete variance. Reporting such tests would convey an inferential rigor the design cannot deliver. The analysis is therefore anchored to effect magnitude and precision rather than to significance thresholds, as recommended for small-sample and case-study research in sports medicine (Hopkins et al., 2009; Ruddock et al., 2019), and is carried out at the individual-athlete level as well as at the group level. Accordingly, no p-values are reported anywhere in this manuscript, and point estimates are accompanied by 95% confidence intervals wherever they can be computed.

#### 2.5.1 Performance data

For each athlete and event (200 m, 400 m, 800 m, 1500 m), the percentage change in race time relative to the baseline time-trial was calculated separately for Competition 1 and Competition 2 as [(Competition time − Baseline time)/Baseline time] × 100%, with negative values denoting faster performance. Interpreting these changes requires a benchmark for the variation an elite swimmer shows between competitions in the absence of any change in fitness — that is, the within-athlete, same-event coefficient of variation between separate competitions. Pyne et al. estimated this quantity directly in Olympic swimmers, modeling 676 official race times from 51 US and Australian swimmers, and reported 0.80% (95% confidence limits 0.73–0.86%) between competitions, as distinct from 0.60% within a single competition (Pyne et al., 2004). We adopted 0.80% because it matches the present comparison in both the quantity estimated and the competitive standard of the cohort and is precisely estimated; a single value was applied to all four events, since no event-specific between-competition estimates have been published for these distances. Multiplying by the conventional factor of 0.3 (Hopkins, 2000; Batterham and Hopkins, 2006) gives a smallest worthwhile change (SWC) of ≈0.24%, against which each athlete-by-event change was classified as SWC-exceeding or as falling within the SWC band. Because the threshold derives from independently published, large-sample data rather than from the present cohort, its validity does not depend on this study’s small N; because the reference CV is nevertheless a modeling choice, the classification was re-run across the full defensible range as a sensitivity analysis (§3.1.1): the confidence limits of the Olympic estimate (SWC 0.22% and 0.26%), the larger national-level variation (CV 1.1% and 1.4%, giving 0.33% and 0.42% (Stewart and Hopkins, 2000)), and the stricter 0.5 × CV medal-probability criterion of Pyne et al. (0.40%). Applying the threshold assumes that the baseline time-trial and the two championships were comparable in the conditions affecting race time. This was not verified: water temperature, pool characteristics, heat-versus-final status, time of day and each athlete’s taper duration were not documented, and venue effects can displace times by amounts comparable to or larger than 0.24% (§4.6). Changes are therefore reported as SWC-exceeding rather than as real.

#### 2.5.2 Hematological and biochemical data

For each athlete and marker, the absolute change from baseline and the relative percentage change — [(Value at time point − Baseline value)/Baseline value] × 100% — were computed at each subsequent sampling point. Standardized paired (within-athlete) effect sizes (Hedges’ g, bias-corrected for small samples) with 95% confidence intervals were calculated for each marker at each post-baseline time point relative to baseline, and interpreted against conventional magnitude thresholds (trivial, < 0.2; small, 0.2–0.6; moderate, 0.6–1.2; large, 1.2–2.0; very large, > 2.0) (Batterham and Hopkins, 2006; Hopkins et al., 2009). Several of these intervals are wide relative to their point estimates, and where an interval spans from a small to an extremely large effect, or includes zero, the direction of change may be reasonably supported while its magnitude remains poorly estimated. Point estimates are therefore described throughout as ‘consistent with’ or ‘compatible with’ a given magnitude, and no marker at any time point is described as demonstrating an established adaptation. Interpretation rests on the magnitude and precision of each effect size together with the individual-athlete trajectories, following case-study reporting guidance in sports science (Halperin, 2018; González-Ravé et al., 2023).

#### 2.5.3 Training-load context

Weekly training intensity distribution (percentage of total volume in Zones 1–3) is reported descriptively alongside the performance and hematological trajectories to provide training context. Because these are single, team-level weekly values rather than independent replicate measurements, they do not constitute a sample suitable for correlational testing; any correspondence between TID phase and physiological or performance change is therefore reported as an observation for future, adequately powered investigation rather than as a statistical association.

#### 2.5.4 Descriptive summaries and reporting

Group data are reported as mean ± SD: race times at each of the three testing points, hematological markers at each of the four sampling weeks, and TID in each macrocycle phase as the mean percentage of total training volume in Zones 1–3. Individual athlete-by-event performance changes are reported in full in a table, in keeping with progressive-statistics recommendations for small-sample sports-science research.

## 3 Results

### 3.1 Swimming performance

The freestyle performance of the swimmers showed a general trend toward improvement across the competitive season (**Table 1**); the group-mean values below are descriptive and are not accompanied by inferential tests, for the reasons set out in §2.5. Compared with the baseline test, group-mean times in Competition 1 were faster by 0.32% (200 m), 1.33% (400 m) and 0.52% (1500 m), and essentially unchanged in the 800 m (0.02%). In Competition 2 the group means were faster than baseline by 0.83% (200 m), 1.17% (400 m), 0.18% (800 m) and 0.50% (1500 m).

**Table 1:** Freestyle performance (mean ± SD) across training and competition phases.

Phase	200 m (s)	400 m (s)	800 m (s)	1500 m (s)
Baseline (TEST)	120.17 ± 1.80	251.95 ± 5.38	512.49 ± 8.98	982.77 ± 13.64
Competition 1	119.78 ± 2.42	248.61 ± 5.82	512.40 ± 9.52	977.65 ± 22.07
Competition 2	119.17 ± 2.31	249.01 ± 5.73	511.56 ± 8.60	977.89 ± 17.16

#### 3.1.1 Smallest-worthwhile-change classification

To provide a benchmark for race-to-race variability, each athlete’s percentage change from baseline was benchmarked against a smallest worthwhile change (SWC) of ≈0.24%, derived as 0.3 × a representative 0.8% between-competition coefficient of variation for elite swimmers (Pyne et al., 2004). Applying this threshold across the 20 athlete-by-event observations, 12 of 20 changes (60%) at Competition 1 and 18 of 20 changes (90%) at Competition 2 exceeded the SWC (**Table 2**). Among the SWC-exceeding changes, the substantial majority were improvements over baseline — 10 of 12 (83%) at Competition 1 and 13 of 18 (72%) at Competition 2 — while the remainder were SWC-exceeding regressions: two at Competition 1 (Swimmer 4, 200 m; Swimmer 2, 800 m) and five at Competition 2, the latter concentrated in the 800 m and 1500 m. The proportion of SWC-exceeding changes rose markedly from Competition 1 to Competition 2 (60% → 90%), so that a greater share of changes exceeded the threshold as the season progressed.

**Table 2:** Individual performance changes relative to baseline, classified against the smallest worthwhile change.

ID	Event	Baseline (s)	Δ Comp 1 (%)	Comp 1 vs SWC	Δ Comp 2 (%)	Comp 2 vs SWC
1	200 m	120.21	-0.05%	< SWC	-0.41%	≥ SWC
	400 m	252.62	-0.84%	≥ SWC	-1.23%	≥ SWC
	800 m	511.78	0.21%	< SWC	0.44%	≥ SWC
	1500 m	988.03	0.14%	< SWC	-2.33%	≥ SWC
2	200 m	122.11	-0.52%	≥ SWC	-0.03%	< SWC
	400 m	254.94	-1.15%	≥ SWC	-1.21%	≥ SWC
	800 m	507.97	1.00%	≥ SWC	0.39%	≥ SWC
	1500 m	977.26	-0.16%	< SWC	0.80%	≥ SWC
3	200 m	117.92	-2.03%	≥ SWC	-1.91%	≥ SWC
	400 m	244.03	-1.67%	≥ SWC	-1.17%	≥ SWC
	800 m	505.43	-0.94%	≥ SWC	-1.27%	≥ SWC
	1500 m	968.59	-2.54%	≥ SWC	-1.36%	≥ SWC
4	200 m	118.89	1.80%	≥ SWC	-0.09%	< SWC
	400 m	249.93	-1.67%	≥ SWC	-1.50%	≥ SWC
	800 m	509.24	-0.13%	< SWC	0.51%	≥ SWC
	1500 m	976.18	-0.05%	< SWC	1.07%	≥ SWC
5	200 m	121.74	-0.93%	≥ SWC	-1.77%	≥ SWC
	400 m	258.24	-1.34%	≥ SWC	-0.74%	≥ SWC
	800 m	528.01	-0.21%	< SWC	-1.00%	≥ SWC
	1500 m	1003.79	-0.03%	< SWC	-0.65%	≥ SWC

Sensitivity to the assumed reference variability. Because the 0.24% threshold rests on a chosen coefficient of variation, the classification was repeated across the range set out in §2.5.1. At the confidence limits of the Olympic between-competition estimate (SWC 0.22% and 0.26% (Pyne et al., 2004)), at the national-level estimates (0.33% and 0.42% (Stewart and Hopkins, 2000)) and at the stricter medal-probability criterion (0.40% (Pyne et al., 2004)), the proportion of SWC-exceeding changes at Competition 1 was 12 of 20 (60%) in every case. At Competition 2 it was 18 of 20 (90%) at thresholds up to 0.33%, 17 of 20 (85%) at 0.40% and 16 of 20 (80%) at 0.42%. The classification is therefore insensitive to the reference value throughout the defensible range at Competition 1 and only mildly sensitive at Competition 2, and the pattern on which the Discussion relies — a higher proportion of SWC-exceeding changes at the second than at the first championship — holds in every case.

#### 3.1.2 Individual response heterogeneity

A key finding was the breakthrough performance of Swimmer 3, whose 400 m freestyle time in Competition 1 improved by 1.67% — approximately seven times the SWC threshold, an unambiguous and non-trivial improvement — and set a new Asian record. Individual adaptation patterns were heterogeneous: Swimmer 3 showed consistent, broad-ranging, SWC-exceeding improvements across all four events without regression; Swimmer 5 progressed from SWC-exceeding gains in two of four events at Competition 1 to SWC-exceeding gains in all four at Competition 2, whereas Swimmer 2 recorded SWC-exceeding gains in the 200 m and 400 m at Competition 1 alongside an SWC-exceeding 800 m regression, and by Competition 2 retained an SWC-exceeding gain only in the 400 m, with SWC-exceeding regressions in the 800 m and 1500 m; Swimmer 4 showed an SWC-exceeding 200 m regression at Competition 1 that had returned to within the SWC band by Competition 2 (−0.09%), alongside SWC-exceeding 400 m improvements at both competitions (−1.67% and −1.50%); and Swimmer 1’s changes were more often within the SWC band at Competition 1 but were predominantly SWC-exceeding improvements by Competition 2. These trajectories describe a season in which most athletes improved and in which the timing of each athlete’s peak differed; with five athletes, no control group and multiple simultaneous interventions, they cannot establish the efficacy of the training cycle itself.

### 3.2 Hematological, muscle-damage, and stress-hormone markers

Oxygen-transport capacity: Red blood cell count (RBC) rose from 4.52 ± 0.09×10¹²/L at baseline to a peak of 4.84 ± 0.18×10¹²/L at Week 27 (paired Hedges’ g = 2.32, 95% CI [0.64, 4.01], very large), remaining elevated at Week 49 (4.81 ± 0.07×10¹²/L, g = 1.71, 95% CI [0.33, 3.08], large). The Week 17 elevation (4.61 ± 0.37×10¹²/L) corresponded to only a small effect (g = 0.26, 95% CI [-0.63, 1.15]), consistent with the first altitude block’s more modest and more variable early erythropoietic response. Hemoglobin (HGB) followed a comparable but more modest trajectory, rising from 136.6 ± 5.0 g/L to 141.4 ± 3.8 g/L at Week 27 (g = 0.43, small) before reaching its largest effect at Week 49 (140.4 ± 4.4 g/L, g = 0.60, moderate). Hematocrit (HCT) showed its largest effect at Week 27 (42.8 ± 0.7%, g = 2.04, 95% CI [0.50, 3.58], very large), regressing to a small effect by Week 49 (42.0 ± 1.7%, g = 0.31).

Muscle damage. Creatine kinase (CK) rose sharply from a rested baseline of 109.6 ± 8.6 U/L to 223.4 ± 60.7 U/L at Week 17 (g = 1.59, 95% CI [0.27, 2.91], large), reflecting substantial muscle microtrauma during the first altitude block and its integration phase. CK then declined progressively — to 177.4 ± 76.0 U/L at Week 27 (g = 0.70, moderate) and 127.2 ± 59.1 U/L at Week 49 (g = 0.26, small) — tracking closely with the scheduled reduction in training volume during each taper and compatible with a lower muscle-damage state at the two pre-competition sampling points.

Stress hormone. Serum cortisol increased from 17.2 ± 0.7 µg/dL at baseline to 21.7 ± 2.5 µg/dL at Week 17 (g = 1.63, 95% CI [0.29, 2.97], large), consistent with accumulated stress from concurrent high-volume and hypoxic loading. Cortisol declined thereafter to 19.7 ± 2.0 µg/dL at Week 27 (g = 1.13, 95% CI [0.01, 2.25], moderate) and fell below baseline by Week 49 (16.4 ± 3.5 µg/dL, g = -0.17, trivial), compatible with a lower physiological stress state at that sampled time point (**Table 3**).

**Table 3:** Hematological, muscle-damage, and stress-hormone markers (mean ± SD) with paired (within-athlete) Hedges’ g effect sizes (95% CI) relative to baseline.

Marker	Baseline (Week 7)	Value	Hedges’ g [95% CI] vs Baseline	Magnitude
Week 17				
RBC (×10¹²/L)	4.52 ± 0.09	4.61 ± 0.37	0.26 [-0.63, 1.15]	small
HGB (g/L)	136.6 ± 5.0	139.2 ± 3.6	0.28 [-0.61, 1.18]	small
HCT (%)	41.2 ± 0.6	41.6 ± 0.6	0.30 [-0.60, 1.19]	small
CK (U/L)	109.6 ± 8.6	223.4 ± 60.7	1.59 [0.27, 2.91]	large
Cortisol (µg/dL)	17.2 ± 0.7	21.7 ± 2.5	1.63 [0.29, 2.97]	large
Week 27				
RBC (×10¹²/L)	4.52 ± 0.09	4.84 ± 0.18	2.32 [0.64, 4.01]	very large
HGB (g/L)	136.6 ± 5.0	141.4 ± 3.8	0.43 [-0.48, 1.35]	small
HCT (%)	41.2 ± 0.6	42.8 ± 0.7	2.04 [0.50, 3.58]	very large
CK (U/L)	109.6 ± 8.6	177.4 ± 76.0	0.70 [-0.28, 1.68]	moderate
Cortisol (µg/dL)	17.2 ± 0.7	19.7 ± 2.0	1.13 [0.01, 2.25]	moderate
Week 49				
RBC (×10¹²/L)	4.52 ± 0.09	4.81 ± 0.07	1.71 [0.33, 3.08]	large
HGB (g/L)	136.6 ± 5.0	140.4 ± 4.4	0.60 [-0.35, 1.55]	moderate
HCT (%)	41.2 ± 0.6	42.0 ± 1.7	0.31 [-0.59, 1.21]	small
CK (U/L)	109.6 ± 8.6	127.2 ± 59.1	0.26 [-0.63, 1.15]	small
Cortisol (µg/dL)	17.2 ± 0.7	16.4 ± 3.5	-0.17 [-1.06, 0.71]	trivial

Interpretive note on the hematological results. Three qualifications apply to the values reported above and should be carried into their interpretation. First, RBC, HGB and HCT are concentration-based indices expressed per unit volume of blood, and they respond to changes in plasma volume as well as to changes in circulating red-cell mass; plasma volume itself is altered by training status, heat, hydration, posture, acute altitude exposure and reproductive-hormone status (Sawka et al., 2000; Stachenfeld and Taylor, 2009). Total hemoglobin mass was not measured by carbon monoxide rebreathing (Schmidt and Prommer, 2005), so an expansion of red-cell mass cannot be distinguished here from a contraction of plasma volume, and the changes reported must not be read as a direct measure of erythropoietic adaptation. Second, several confidence intervals are wide relative to their point estimates — the Week 27 RBC interval, for instance, spans from a moderate to an extraordinary effect — so the magnitude of these effects is imprecisely estimated even where the direction is reasonably supported. Third, all values are group means from five athletes whose iron status, menstrual-cycle phase and hormonal contraceptive use were unknown at each draw, and each of these can shift concentration-based hematological indices and cortisol independently of training (Kirschbaum et al., 1999; Govus et al., 2015; Sim et al., 2019; McNulty et al., 2020; Elliott-Sale et al., 2021).

### 3.3 Training intensity distribution

Training intensity distribution was quantified across the 52-week macrocycle to characterize the periodization strategies employed for the two national championships. Phase 1 (Weeks 1–19) followed a pyramidal model, with weekly volume increasing from ≈38 km to a peak of ≈59 km by Week 15; Zone 1 comprised 48–54% of weekly volume, Zone 2 30–36%, and Zone 3 remained stable at 15–16%. Phase 2 (Weeks 20–29) began with a distinct shift to a polarized model during the second altitude camp (2,400 m, Weeks 20–25): Zone 1 rose to 72–74%, Zone 2 fell to 7–8%, and Zone 3 reached 18–20%. A brief pyramidal taper (Week 26) was followed by a threshold-oriented block (Weeks 27–28, Zone 2 = 42–43%) and a polarized competition week (Zone 1 = 78.3%, Zone 2 = 4.7%, Zone 3 = 17.0%). Phase 3 (Weeks 30–52) opened with a pyramidal transition week (Week 30) and a sustained threshold block (Weeks 31–35, Zone 2 = 41–43%), transitioned to an extended polarized block (Weeks 36–47; Zone 1 = 72–75%, Zone 2 = 5–8%, Zone 3 = 18–20%) that was adopted two weeks before departure and encompassed the third and longest altitude camp (1,890 m, Weeks 38–47), and concluded with a progressive pyramidal-to-polarized taper (Weeks 48–51) culminating in a polarized final test week (Zone 1 = 79.1%, Zone 2 = 4.1%, Zone 3 = 16.8%) (**Figures 2**, **3**).

Training-load context for the hematological trajectory. The weekly training log allows the training context of each blood draw to be described directly rather than inferred. Week 27 — the taper into Competition 1 — carried both the highest weekly training volume of the four sampling weeks and the largest threshold (Zone 2) emphasis and corresponds to the largest RBC and HCT effect sizes reported in §3.2 (**Table 3**). Week 49, the taper into Competition 2, carried a pyramidal, Zone-1-dominant distribution at lower relative volume, and corresponds to the smallest cortisol effect size (g = -0.17, trivial) of the four sampling weeks. These four data points are too few to support a correlation coefficient or any other quantitative association statistic — a formal analysis of this kind would need each athlete’s individual training exposure logged against their individual outcome trajectory, not the single team-level weekly record available here — so the pattern above is offered as descriptive context for the case narrative, not as statistical evidence of a training–hematology relationship.

## 4 Discussion

### 4.1 Double-peak periodization and dynamic training-intensity distribution

The 52-week macrocycle was deliberately structured around two national championships, with three altitude camps and matching sea-level integration and taper phases – a scenario less documented than single-peak preparations (González-Ravé et al., 2021; Hermosilla et al., 2021). The progressive increase in the proportion of athlete-by-event changes exceeding the smallest worthwhile change (from 60% at Competition 1 to 90% at Competition 2) is consistent with a cumulative rather than conflicting adaptation, although causality cannot be inferred. A distinctive feature was the phase-dependent switching between pyramidal, threshold and polarized training-intensity distribution (TID) models. The basal development phase used a pyramidal distribution, altitude blocks were paired with a polarized pattern (Zone 2 reduced to 5–8%), and the pre-competition sharpening phases emphasized threshold work (Zone 2 ~41–43%). This sequential use contrasts with previous head-to-head comparisons (Clemente-Suárez et al., 2017; Pla et al., 2019; Arroyo-Toledo et al., 2021) and suggests that the relative effectiveness of each TID model depends more on its placement within the season than on an intrinsic superiority. The polarized blocks occurred concurrently with altitude exposure, consistent with the reported benefits of polarized training (Stöggl and Sperlich, 2014; Silva Oliveira et al., 2024), but the altitude and TID effects cannot be disentangled here. Practically, coaches may consider flexible TID switching across a multi-peak season, provided that internal load is monitored.

### 4.2 Altitude exposure and hematological changes: association, not causation

The observed elevations in concentration-based red blood cell count (RBC) and hematocrit (HCT) following the altitude camps are directionally consistent with previous reports of increased hemoglobin mass after similar hypoxic stimuli (Astridge et al., 2024; Mujika et al., 2024; Carin et al., 2025). However, several unmeasured confounders preclude a causal attribution. First, total hemoglobin mass was not measured by carbon monoxide rebreathing (Schmidt and Prommer, 2005); therefore, the concentration increases could partly reflect plasma-volume contraction rather than erythropoiesis. Second, iron status (ferritin, transferrin saturation) and supplementation were not recorded, although iron availability is a critical determinant of the erythropoietic response to altitude and is particularly relevant in female endurance athletes (Govus et al., 2015; Sim et al., 2019). The 2.7-fold inter-individual spread in RBC response observed here may therefore be largely explained by differential iron status rather than by training responsiveness. Third, menstrual-cycle phase and hormonal contraceptive use, which affect plasma volume and cortisol dynamics (Kirschbaum et al., 1999; Sawka et al., 2000; Stachenfeld and Taylor, 2009), were not documented – a gap that weakens interpretation of all hematological and endocrine measures. Furthermore, the magnitude of hematological change did not track performance improvement, echoing findings that hemoglobin-mass gains are not significantly correlated with race outcomes in elite swimmers (Mujika et al., 2024). Thus, while the hematological trajectory is compatible with an altitude effect, it should be read as an association only (Lundby et al., 2012).

Because all five athletes were female, the absence of menstrual and hormonal data constitutes an endocrinological confound rather than a documentation gap, and it operates through three distinct channels. Estradiol and progesterone modulate plasma-volume expansion, so an unrecorded difference in cycle phase between two draws can shift RBC, HGB and HCT in either direction independently of red-cell mass (Sawka et al., 2000; Stachenfeld and Taylor, 2009). Free cortisol and hypothalamic–pituitary–adrenal responsiveness differ systematically between the follicular and luteal phases and are further modified by hormonal contraceptive use, largely through estradiol-induced changes in corticosteroid-binding globulin (Kirschbaum et al., 1999); consequently, the cortisol trajectory reported in §3.2 is confounded by an unmeasured endocrine variable acting on the same measure with a comparable magnitude. At the performance level, a network meta-analysis of 78 studies found at most a trivial average reduction in the early follicular phase, with substantial between-study heterogeneity and a recommendation for individual rather than group-level interpretation (McNulty et al., 2020). Because the four sampling weeks were fixed by the training calendar rather than by cycle phase, sampling could coincide non-randomly with particular phases across athletes, making this a potential systematic bias rather than merely additional noise. In the context of a longitudinal observation of a routine squad training program, the present design does not allow for phase-anchored sampling or hormonal-status standardization (Elliott-Sale et al., 2021); this is a constraint inherent to real-world elite-athlete monitoring rather than an avoidable methodological omission, and it should be considered when interpreting the endocrine and hematological findings.

### 4.3 Creatine kinase and cortisol trajectories: recovery status versus sampling resolution

Creatine kinase (CK) and cortisol showed their largest elevations at the mid-season sampling point (Week 17) and declined toward baseline by Week 49, which is compatible with effective load reduction during the tapers (Hellard et al., 2017; Mujika et al., 2018). Nevertheless, the four sampling time points across 52 weeks provide only a phase-level snapshot. CK kinetics (peak 24–72 h after damaging exercise) and cortisol’s strong circadian and day-to-day variability (Clarkson and Hubal, 2002) mean that the observed values describe rested states on four specific Mondays, not the continuous profile of muscle damage or neuroendocrine stress. Peaks occurring between sampling weeks – for instance, during the highest-load weeks of Altitude III – remain invisible. Hence, statements about ‘effective recovery’ should be restricted to the sampled time points. Future studies should sample at least monthly, with additional draws bracketing each altitude block and taper, to capture the true kinetics of these fast-turnover markers.

### 4.4 Sources of individual heterogeneity and unmeasured confounders

A key observation is the substantial inter-individual variation in both performance and biomarker responses to the same program – from Swimmer 3’s Asian-record breakthrough to Swimmer 4’s event-specific regression and rebound. This heterogeneity is often averaged out in group designs but is preserved in the case-series approach. Several candidate mechanisms can be proposed: baseline competitive standard, responder/non-responder status to hypoxia, iron availability, event specialization, training age, energy availability, and menstrual status (Govus et al., 2015; Sim et al., 2019; McNulty et al., 2020; Elliott-Sale et al., 2021; Mountjoy et al., 2023). However, none of these was measured in this study. In particular, the absence of serial ferritin, hepcidin, and supplementation records makes it impossible to attribute the hematological spread to altitude response versus iron status. Likewise, unrecorded menstrual-cycle phase constitute a major confound for both hematocrit (via plasma volume) and cortisol (Kirschbaum et al., 1999; Sawka et al., 2000; Stachenfeld and Taylor, 2009), and could systematically bias the four fixed sampling weeks. Similarly, dryland training was not quantified, so CK elevations and even some performance gains may be partly due to resistance work rather than in-water periodization (Clarkson and Hubal, 2002; Mujika et al., 2018). Therefore, while the data clearly demonstrate that a nominally identical program yields divergent individual outcomes, they cannot explain why.

Two components of the athletes’ total training load lay outside the monitoring framework, and both bear directly on the attributions discussed above. The squad’s dryland strength-and-conditioning program ran throughout the macrocycle but was delivered outside the research protocol and was not logged in quantifiable form; consequently, every training-load descriptor in this manuscript refers exclusively to in-water swimming and represents a lower bound on total load. Because creatine kinase is a general marker of muscle-membrane disruption and responds strongly to eccentric and unaccustomed resistance work (Clarkson and Hubal, 2002), the near-doubling at Week 17 is equally compatible with a concurrent dryland block; and season-long gains in starts, turns and force application would appear in the race times indistinguishably from an effect of the in-water periodization (Mujika et al., 2018). Internal load was likewise not captured: no session-RPE, resting heart rate or heart-rate variability was collected, despite consensus guidance recommending that external and internal indices be paired, precisely because identical prescriptions impose different strain depending on fatigue, hypoxia, illness and sleep (Bourdon et al., 2017). This distinction is most consequential at altitude, where a velocity classified as Zone 1 at sea level may impose materially greater internal demand; the intensity distribution reported here is therefore a record of prescribed and completed in-water work rather than a validated measure of physiological strain. For the same reason, the present monitoring framework cannot distinguish whether a blunted hematological response reflects under-production of erythropoietin, iron limitation, or a loss of training quality at 2,400 m – three mechanisms with entirely different practical remedies. This is a boundary of the available measurement layer, not a procedural shortcoming, and it should inform the interpretation of both the training-load and the hematological findings.

### 4.5 Proposed physiological pathway: framework, not demonstrated mechanism

**Figure 4** provides an integrated visual summary of the observed variables: panel (a) maps the training phases, dominant TID models, weekly in-water volume, blood-sampling weeks, and the two championship time points; panels (b) and (c) display the group-mean percentage changes from baseline for oxygen-transport indices and for cortisol, respectively (noting that these are concentration-based and therefore reflect plasma-volume as well as erythropoietic influences); panel (d) presents the paired Hedges’ g effect sizes with 95% confidence intervals for all markers at each post-baseline sampling week (where interval width is the primary information to read); and panel (e) summarizes the classification of the 20 athlete-by-event performance changes against the SWC at each championship. A coherent pathway can be outlined: hypoxic stimulus → erythropoietin-mediated red-cell mass expansion and peripheral mitochondrial adaptations → increased sustainable velocity, expressed through a neuromuscular system restored by tapering (Chapman et al., 1998; Gore et al., 2013; Bishop et al., 2014; Mujika et al., 2018). However, erythropoietin, total hemoglobin mass, VO_2_max, muscle biopsy data, and neuromuscular function were not assessed. The pathway therefore remains a theoretical framework imported from the literature, not a mechanism demonstrated by the present data. **Figure 4** should be read as a visual synthesis of what was actually measured, with the unmeasured mechanistic links — marked conceptually in the text — remaining to be addressed in future investigations.

### 4.6 Consolidated limitations and future directions

The constraints discussed above are cumulative: no control group, no randomization, N = 5, four biological samples across 52 weeks, a bundled intervention (TID switching, three altitude exposures, two tapers, technical work, unquantified dryland training), and unmeasured confounders (iron status, menstrual phase, plasma volume, internal load). Under these conditions, no causal claim linking the periodized plan to performance improvements is defensible. The study’s value lies in its detailed, ecologically valid description of a real-world double-peak season in an elite squad, analyzed at the individual level with a statistical framework calibrated to what five athletes can support.

For future research, we recommend: (i) monthly blood sampling with additional points around altitude and taper blocks; (ii) direct measurement of total hemoglobin mass by CO rebreathing (Schmidt and Prommer, 2005); (iii) serial ferritin and hepcidin monitoring, with standardized iron supplementation (Govus et al., 2015); (iv) documentation of menstrual-cycle phase and hormonal contraceptive use, with phase-anchored testing where feasible (Elliott-Sale et al., 2021); (v) quantification of dryland training and use of session-RPE or heart-rate variability for internal load (Foster et al., 2001; Plews et al., 2013; Bourdon et al., 2017); (vi) individual-level (not team-level) logging of training exposure to allow valid exposure-outcome associations; and (vii) recording of competition-environment variables (water temperature, pool characteristics, time of day) to contextualize single-race variability. Pooling comparable case series across seasons or centers, while preserving individual-level analysis, would also enhance generalizability.

## 5 Conclusion

This collective case study provides a detailed, ecologically valid characterization of a 52-week double-peak macrocycle in elite female middle- and long-distance swimmers, integrating weekly training-intensity distribution, three successive altitude camps, and two timed tapers. Across the season, the majority of individual athlete-by-event performance changes exceeded the smallest worthwhile change derived from competition-to-competition variability, with the proportion of SWC-exceeding changes increasing from 60% at the first national championship to 90% at the second. The phase-dependent shifting between pyramidal, polarized, and threshold intensity distributions, aligned with the functional objectives of each mesocycle, was accompanied by a progressive normalization of creatine kinase and cortisol and by sustained elevations in concentration-based erythrocyte indices – a pattern accompanying the progression of the macrocycle.

Nevertheless, the study’s design – lacking a control group, relying on concentration-based rather than mass-based hematology, and omitting serial iron biomarkers, menstrual-cycle phase, dry-land load, and internal-load verification – precludes any causal attribution of the observed performance gains to the periodized plan itself. Marked inter-individual heterogeneity further cautions against uniform application of altitude or intensity-distribution strategies. The contribution is therefore descriptive and hypothesis-generating: it offers a transparent, individual-level case reference for practitioners managing multi-peak seasons and provides a methodological blueprint for future adequately powered investigations that should incorporate individual-level training logs, direct quantification of erythropoietic adaptation, and phase-anchored female-specific physiological monitoring.

## Data Availability

The raw data supporting the conclusions of this article will be made available by the authors, without undue reservation.
